# Adverse drug reactions and kinetics of cisplatin excretion in urine of patients undergoing cisplatin chemotherapy and radiotherapy for head and neck cancer: a prospective study

**DOI:** 10.1186/s40199-017-0178-9

**Published:** 2017-04-24

**Authors:** Marília Berlofa Visacri, Eder de Carvalho Pincinato, Graziele Baldan Ferrari, Júlia Coelho França Quintanilha, Priscila Gava Mazzola, Carmen Silvia Passos Lima, Patricia Moriel

**Affiliations:** 1 School of Medical Sciences (FCM), University of Campinas (UNICAMP), Tessália Vieira de Camargo, 126, Cidade Universitária “Zeferino Vaz”, Zip Code 13083-887 Campinas, SP Brazil; 2 Department of Biological and Health Science Center, Mackenzie Presbyterian University, Rua da Consolação 896, Consolação, Zip Code 01302-907 São Paulo, SP Brazil; 3 Faculty of Pharmaceutical Sciences (FCF), University of Campinas (UNICAMP), Cândido Portinari, 200, Cidade Universitária “Zeferino Vaz” - Barão Geraldo, Zip Code 13083-871 Campinas, SP Brazil

**Keywords:** Adverse drug reaction, Excretion, Urine, Cisplatin, Chemotherapy, Head and neck cancer

## Abstract

**Background:**

Cisplatin is a high-potency anticancer agent; however, it causes significant adverse drug reactions (ADRs). Potential pharmacokinetic markers must be studied to predict or prevent cisplatin-induced ADRs and achieve better prognosis. This study was designed to investigate the relationship between ADRs and kinetics of cisplatin excretion in the urine of patients undergoing high-dose cisplatin chemotherapy and radiotherapy for head and neck cancer.

**Methods:**

Outpatients with head and neck cancer received a first cycle of high-dose cisplatin chemotherapy (80–100 mg/m^2^) concurrent to radiotherapy. ADRs (haematological, renal, and gastrointestinal reactions) were classified based on severity by National Cancer Institute Common Terminology Criteria for Adverse Events (CTCAE, version 4, grade 0–4). The kinetics of cisplatin excretion in urine was evaluated by high-performance liquid chromatography over three time periods: 0–12, 12–24, and 24–48 h after the administration of cisplatin. Spearman Correlation test and regression analysis were performed to assess the relationship between ADRs and cisplatin excretion in the urine.

**Results:**

In total, 59 patients with a mean age of 55.6 ± 9.4 years were analysed; most patients were male (86.4%), white (79.7%), and with pharyngeal tumours in advanced stages (66.1%). The most frequently observed ADRs were anaemia (81.4%), lymphopenia (78%), and nausea (64.4%); mostly grades 1 and 2 of toxicity. The mean cisplatin excretion was 70.3 ± 64.4, 7.3 ± 6.3, and 5 ± 4 μg/mg creatinine at 0–12, 12–24, and 24–48 h, respectively. Statistical analysis showed that the amount of cisplatin excreted did not influence the severity of ADRs.

**Conclusions:**

The most frequent ADRs were anaemia, lymphopenia, and nausea. Grades 1 and 2 were the severities for most ADRs. The period over which the highest cisplatin excretion observed was 0–12 h after chemotherapy, and cisplatin excretion could not predict toxicity.

**Graphical abstract:**

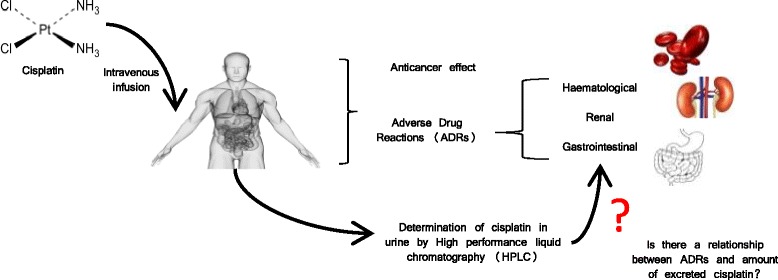

## Background

Head and neck squamous cell carcinomas (HNSCC) are malignant tumours located in the upper aerodigestive tract, and the most commonly affected sites are the oral cavity, pharynx, and larynx [1]. The best treatment for advanced HNSCC when surgery is contraindicated includes chemoradiation, i.e. chemotherapy with a platinum-based drug along with conventional radiotherapy. Chemoradiation increases patient survival of 5 years by 8% and lowers mortality risk by 19% compared with radiotherapy alone [2]. Available and validated literature recommends the treatment based on high-dose cisplatin chemotherapy (100 mg/m^2^) every 3 weeks along with conventional radiotherapy [3, 4]. However, chemoradiation has a high risk for severe toxicity.

The most commonly used platinum derivative is cisplatin. It is a complex containing a central atom of platinum surrounded by two chlorine atoms and two ammonia groups. Its cytotoxic action is analogous to that of alkylating agents. When entering the cell, the chloride ion dissociates, leaving a reactive complex that reacts with water and then interacts with the DNA by forming covalent bonds, preferably at the N7 position of adenine and guanine. The reaction at two different DNA sites produces intrachain (>90%) or interchain (<5%) bonds. These platinum-DNA complexes can inhibit DNA synthesis and consequently its transcription, which leads to the induction of apoptosis in tumour cells [5]. Furthermore, cisplatin binds to mitochondrial DNA that inhibits adenosine triphosphate (ATP) production, reduces ATPase activity, changes intracellular calcium content, and decreases the rate of cellular respiration, which results in the production of reactive oxygen species and cellular lipid peroxidation [6].

After intravenous administration, 90% of the cisplatin binds with plasma proteins such as albumin, gammaglobulin, and transferrin [7] and is distributed to the tissues, particularly the kidney, liver, and prostate [8]. The formation of conjugates between glutathione and cisplatin, through the action of glutathione-S-transferase, is an important step in the inactivation and elimination of cisplatin [9, 10]. Cisplatin is primarily excreted by the kidneys [11]. In a study of the administration of radioactive cisplatin, urinary elimination was incomplete with 25–45% of the radioactivity decay in the first 5 days. Furthermore, the level of radioactive decay occurred in a biphasic manner: half-life varied from 25 to 49 min and from 58 to 73 h in the initial and terminal phases, respectively [7].

As described, cisplatin is a high-potency anticancer agent with favourable pharmacokinetics. However, it has been noted that, similar to other antineoplastic agents, it causes significant adverse drug reactions (ADRs) such as myelosuppression, emesis, and nephrotoxicity. It is necessary to study potential pharmacokinetic and/or genetic markers to predict or prevent ADRs and achieve a better clinical outcome. Pharmacokinetic studies are usually performed to determine the drug concentration in the blood; however, there is a good correlation between the cisplatin concentration in the blood and urine, indicating that both methods can be used in the pharmacokinetic studies of cisplatin [12]. Regarding the intracellular concentration of cisplatin, a correlation between cisplatin concentration in the plasma and formation of cisplatin-DNA adducts in leukocytes of cancer patients is controversial [13, 14].

Studies designed to investigate the association between cisplatin excretion in urine and its ADRs are scarce. Therefore, the current study aimed to investigate the relationship between ADRs and the kinetics of cisplatin excretion in the urine of patients undergoing high-dose chemotherapy and radiotherapy for head and neck cancers.

## Methods

### Study design

This was a prospective study, with consecutive sampling performed from May 2011 to January 2013, conducted at the Clinical Oncology department of a teaching hospital in São Paulo, Brazil. The Research Ethics Committee of the institution approved this study and all patients signed a consent form authorising the use of their data (number 274/2011, CAAE: 0218.0.146.000-11).

### Eligibility criteria

The study included patients with HNSCC (primary tumour) and on high-dose cisplatin chemotherapy (80–100 mg/m^2^) with concurrent radiotherapy. Patients who had undergone tumour treatment in the past (surgery, chemotherapy, or radiotherapy), those with severe psychiatric problem, those who used nephrotoxic drugs, or those who refused to participate were excluded.

### Treatment protocols

Chemotherapy comprised three cycles of chemotherapy with high-dose (80–100 mg/m^2^) cisplatin on days 1, 22, and 43. In this study, we investigated only the first cycle of chemotherapy. On each day of chemotherapy, the patients received vigorous hydration (3 L of saline solution 0.9%), diuretics (125 mL of mannitol 20%), electrolytes (20 mL of potassium chloride 19.1% and 10 mL of magnesium sulphate 10%), and prophylaxis of acute emesis (20 mg of dexamethasone plus 24 mg of ondansetron). For the treatment and prevention of delayed emesis, the patients received 10 mg metoclopramide every 6 h and 8 mg dexamethasone every 12 h for three consecutive days after the chemotherapy sessions.

Concomitant with the three cycles of chemotherapy, the patients received a total dose of 70 Gy of radiation therapy divided into 35 daily applications of 2 Gy administered for 5 days per week for 7 weeks. Radiation was performed using cobalt-60 (Alcyon II, GE, France) and a linear accelerator (6 MV) (Varian Medical Systems, CA, USA).

### Demographic and clinical data

Data about patient characteristics were obtained from medical records and interviews with patients, including information about age, gender, race, Karnofsky Performance Status (KPS), smoking and drinking categories based on the studies by Jindal et al. [15] and Whitcomb et al. [16], and on the site and stage of tumour [17].

### Adverse drug reactions

ADRs investigated were haematological (anaemia, leukopenia, neutropenia, lymphopenia and thrombocytopenia), renal (change in serum creatinine and creatinine clearance) and gastrointestinal reactions (nausea, vomiting and diarrhoea).

The blood samples of all the patients were collected before (at least 1 week) and after chemotherapy (15–20 days later) for the evaluation of haematological and renal reactions. The values of haemoglobin, leukocytes, neutrophils, lymphocytes, platelets and serum creatinine were obtained. Creatinine clearance was estimated using the Cockroft–Gault formula based on the weight, age and serum creatinine levels of the patient. The values were also used for the classification of ADRs in relation to severity, according to the National Cancer Institute Common Terminology Criteria for Adverse Events (CTCAE, version 4) [18]. Anaemia is common before the beginning of treatment in cancer patients; thus, for the subjects whose haemoglobin levels were below normal before treatment, the toxicity after treatment was classified as grade 1 when the reduction of haemoglobin was ≥1 g/dL from the baseline, considering significant variation. When the baseline values of lymphocytes and creatinine clearance were not normal, toxicity was considered when the reduction was ≥10% from the baseline, considering the biological variation [19, 20] and 1% of balance variation.

The severity of gastrointestinal reactions was also classified according to CTCAE [18], considering the higher grade the symptom reached on Day 1 (first 24 h after chemotherapy–acute phase) to Day 5 (24–120 h after chemotherapy–delayed phase). The number of patients with reactions on each day of occurrence (Day 1–5) was evaluated.

### Kinetics of cisplatin excretion in urine

The 24-h urine samples of the patients were collected in urine collection bottles (2.5 L polypropylene bottle) by voluntary urination during three distinct periods (first period: 0–12 h, the second period: 12–24 h and last period: 24–48 h) after the administration of cisplatin. Following the collection, the sample was homogenised and an aliquot of 45 mL was stored in falcon tube at −80 °C until analysis [21, 22].

For cisplatin urine detection, the previously described procedure was followed [23] with some modifications: In 9 mL of urine, 90 μL of nickel chloride (300 μg/mL) was added (Merck, Darmstadt, Germany) as internal standard and 1 mL of diethyldithiocarbamate (DDTC 10% in 0.1 M NaOH) (Sigma-Aldrich, India) was added for derivatizing. The sample was homogenised and left at 25 °C for 1 h. Thereafter, 1 mL of chloroform was added (Merck, Darmstadt, Germany) in each tube. The tubes were centrifuged for 10 min at 3500 rpm. After centrifugation, the tubes were gently homogenised and re-centrifuged at 3500 rpm for 10 min. Following centrifugation, the supernatant was discarded and the chloroform phase was transferred into a vial for HPLC and subsequent analysis.

The analysis was performed using an HPLC Separation Module system (Waters 2695, USA) with dual absorbance detector (UV-visible detector, wavelength 254 nm) (Waters 2487, USA) and a Hypersil ODS C18 column (150 mm × 4 mm and 4 μm of particle size, Thermo, USA). The HPLC conditions and mobile phase used were according to a method described by Lopez-Flores et al. [24]. The limit of detection was 0.15 μg/mL, the limit of quantification was 0.45 μg/mL, the limit of linearity was 150 μg/mL, and the variation coefficient was 3.40% for the HPLC method.

To normalise the cisplatin values quantified in the urine, urinary creatinine level was determined using a colorimetric/kinetic method (modified Jaffe reaction; Creatinine Laborclin kit®, Paraná, Brazil). Urine was pre-diluted (1:50) and the kit was used to determine urinary creatinine.

### Statistical analysis

Statistical analysis was performed using the Statistics Program for Social Sciences for Windows (SPSS 16.0, SPSS Inc., Chicago, IL, USA) and Statistical Analysis System for Windows (SAS 9.4. SAS Institute Inc., 2002–2008, Cary, NC, USA). The significance level for all analyses was 5% (*p* < 0.05). Continuous and categorical data were described as mean and percentage, respectively. Wilcoxon test (paired samples) and ANOVA with repeated measures were used to analyse changes between assessments (different times). The Spearman Correlation test was used to check the linear correlation between the kinetics of urinary excretion of cisplatin and severity of ADRs and also between ADRs. For correlations that were statistically significant (*p* < 0.05), we considered only correlation coefficient (R) ≥ 0.5 and R ≤ −0.5. We also used univariate linear and logistic regression analyses to check the association between the kinetics of urinary cisplatin excretion and severity of ADRs.

## Results

Of the recruited 95 patients, 36 patients were withdrawn from the study before initiation of the treatment, mainly due to change in the chemotherapy protocol (*n* = 18) and death (*n* = 7), and 59 patients were a part of the study. Demographics and clinical data of the patients are presented in Table 1. The mean age of the patients was 55.6 ± 9.4 years. The patients received a mean dose of 153.6 ± 29.2 mg of cisplatin in the first chemotherapy cycle.

**Table 1:** Demographics and clinical data of study patients (*N* = 59)

Demographics and clinical characteristics	N (%)
Gender	
Men	51 (86.4)
Women	8 (13.6)
Race	
White	47 (79.7)
Non-white	12 (20.3)
Smoking category	
Non-smokers	03 (5.3)
Light smokers	0 (0.0)
Moderate smokers	07 (12.3)
Heavy smokers	47 (82.4)
Not evaluated	02 (3.4)
Drinking category	
Abstainers	08 (14.8)
Light drinkers	03 (5.5)
Moderate drinkers	08 (14.8)
Heavy drinkers	20 (37.1)
Very heavy drinkers	15 (27.8)
Not evaluated	05 (8.5)
KPS	
100	16 (27.1)
90	19 (32.2)
80	11 (18.7)
70	13 (22.0)
Tumour site	
Pharynx	39 (66.1)
Larynx	14 (23.7)
Oral cavity	6 (10.2)
T Stage	
T1	4 (6.8)
T2	10 (16.9)
T3	19 (32.2)
T4	26 (44.1)
N Stage	
N0	12 (20.4)
N1	18 (30.5)
N2	19 (32.2)
N3	10 (46.9)
Stage	
I	1 (1.7)
II	2 (3.4)
III	16 (27.1)
IV	40 (67.8)

*KPS* Karnofsky Performance Status, *N* absolute number, *SD* standard deviation. No patient had distant metastases

Smoking category was classified based on the study by Jindal et al. [15]. Non-smokers were patients that denied having ever smoked; light, moderate and heavy smokers were smokers and exsmokers, and they were classified according to the smoking index (SI), which was the product of the average number of cigarettes smoked per day and the duration of smoking in years; light (SI = 1 – 100), moderate (SI = 101 – 300) and heavy (SI ≥ 301) smokers. Drinking category based on the study by Whitcomb et al. [16]. Average weekly alcohol intake during the maximum lifetime drinking period (drinks/week): abstainers, no alcohol use or <20 drinks in lifetime; light drinkers, ≤3 drinks/week; moderate drinkers, 4–7 drinks/week for females and 4–14 drinks/week for males; heavy drinkers, 8–34 drinks/week for females and 15–34 drinks/week for males; very heavy drinkers, ≥35 drinks/week

Haematological parameters and renal function were analysed for evaluating ADRs. The values of baseline and after the first chemotherapy cycle are presented in Table 2. After chemotherapy with cisplatin, all the parameters changed significantly. However, the mean values for haemoglobin, lymphocytes, and creatinine clearance were below normal.

**Table 2:** Haematological parameters and renal function before and after first chemotherapy cycle with high-dose cisplatin

Parameters	Reference values^a^	Mean ± SD		*p* value^b^
		Baseline	After 1st cycle	
Haematological				
Haemoglobin (g/L)	14–18 (men); 12–16 (women)	12.5 ± 1.7	10.7 ± 1.8	<0.0001
Leukocytes (×10^3^/mm^3^)	4.0–10.0	9.9 ± 3.6	4.9 ± 2.4	<0.0001
Neutrophils (×10^3^/mm^3^)	2.0–8.0	6.5 ± 2.7	3.1 ± 2.0	<0.0001
Lymphocytes (×10^3^/mm^3^)	1.0–4.0	2.0 ± 0.7	0.7 ± 0.4	<0.0001
Platelets (×10^3^/mm^3^)	150.0–400.0	332.6 ± 144.4	248.1 ± 105.1	<0.0001
Renal				
Creatinine (mg/dL)	<1.20 (men); <0.90 (women)	0.8 ± 0.2	1.0 ± 0.4	<0.0001
Creatinine clearance (mL/min)	>90.0	84.0 ± 25.2	69.3 ± 26.2	<0.0001

^a^Reference values of the studied institution. ^b^Comparison of the parameters before and after (variation) the 1st cycle (Wilcoxon test – paired samples). *SD* standard deviation

The ADRs listed by patients were also studied in relation to its severity and classified as grade 0–4 as shown in Table 3. The most frequent ADRs after the first chemotherapy cycle with high-dose cisplatin were anaemia, lymphopenia, and nausea. Moreover, most of the ADRs were grades 1 and 2.

**Table 3:** Frequency and severity of adverse drug reactions among study patients

		Grade - N (%)				
ADRs		1	2	3	4	Patients with ADRs
Haematological	Anaemia	29 (49.2)	16 (27.1)	3 (5.1)	0 (0.0)	48 (81.4)
	Leukopenia	12 (20.3)	9 (15.3)	1 (1.7)	2 (3.4)	24 (40.7)
	Neutropenia	7 (11.8)	4 (6.8)	4 (6.8)	2 (3.4)	17 (28.8)
	Lymphopenia	8 (13.6)	23 (39.0)	13 (22.0)	2 (3.4)	46 (78.0)
	Thrombocytopenia	11 (18.6)	0 (0.0)	0 (0.0)	1 (1.7)	12 (20.3)
Renal	Increase in serum creatinine	11 (19.0)	5 (8.6)	0 (0.0)	0 (0.0)	17 (27.6)
	Reduction in creatinine clearance	14 (24.6)	20 (35.1)	2 (3.5)	0 (0.0)	36 (63.2)
Gastrointestinal	Nausea	13 (22.0)	18 (30.5)	7 (11.9)	-	38 (64.4)
	Vomiting	11 (18.6)	9 (15.3)	8 (13.6)	0 (0.0)	28 (47.5)
	Diarrhoea	5 (8.5)	2 (3.4)	1 (1.7)	0 (0.0)	8 (13.6)

*ADRs* Adverse Drug Reactions, *N* absolute number of patients

Frequencies of nausea, vomiting, and diarrhoea were also studied as a function of the days of occurrence: nausea, day 1: *n* = 17 (28.8%), day 2: *n* = 28 (44.1%), day 3: *n* = 30 (50.8%), day 4: *n* = 26 (44.1%), day 5: *n* = 26 (44.1%); vomiting, day 1: *n* = 8 (13.6%), day 2: *n* = 17 (28.8%), day 3: *n* = 20 (33.9%), day 4: *n* = 16 (27.1%), day 5: *n* = 14 (23.7%); diarrhoea, day 1: *n* = 3 (5.1%), day 2: *n* = 4 (6.8%), day 3: *n* = 2 (3.4%), day 4: *n* = 1 (1.7%), day 5: *n* = 3 (5.1%). Nausea and vomiting were more frequent in the delayed phase (mainly day 2 and 3), but diarrhoea did not have any such difference.

**Fig 1:**
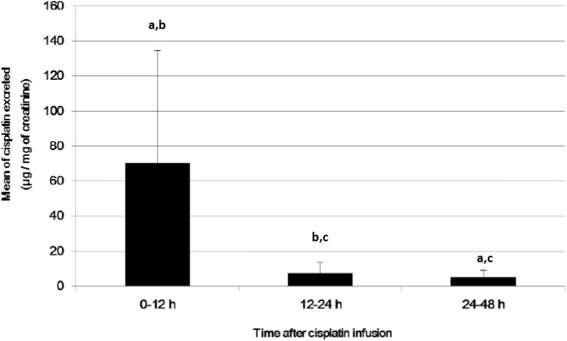
Kinetics of cisplatin excretion in urine. **a**: Statistically significant compared with results for 12–24 h (ANOVA with repeated measures, *p* < 0.0001). **b**: Statistically significant compared with results for 24–48 h (ANOVA with repeated measures, *p* < 0.0001). **c**: Statistically significant compared to period 0–12 h (ANOVA with repeated measures, *p* < 0.0001). *N* = 53 (these analyses could not be done for 6 patients as urine was not collected or collected wrongly). Excreted cisplatin over 0–48 h (sum of three periods) = 82.5 ± 66.5 μg of cisplatin/mg of creatinine

The kinetics of urinary excretion of cisplatin/urine creatinine was analysed over three different periods (0–12 h, 12–24 h and 24–48 h) (Fig. 1). It was observed that patients excreted a significantly higher amount of cisplatin in the first 12 h after chemotherapy.

As expected, significant correlations [severity by grade and number variations (range)] were observed between related ADRs: leukopenia and neutropenia (grade, *R* = 0.739, *p* < 0.001; range, *R* = 0.894, *p* < 0.001); increased creatinine and reduced creatinine clearance (grade, *R* = 0.636, *p* < 0.001; range, *R* = 0.893, *p* < 0.001) and nausea and vomiting (grade, *R* = 0.619, *p* < 0.001).

ADRs [severity by grade and number variations (range)] had no significant correlation with the elimination of cisplatin in the urine (0–12 h, 12–24 h and 24–48 h) (Table 4). When the univariate linear and logistic regression analyses were performed to evaluate the same relationship, no significant result was obtained (Table 4).

**Table 4:** Relationship between ADRs and cisplatin excretion in urine

	Cisplatin excretion					
	0–12 h		12–24 h		24–48 h	
	SCT (R/*p* value)	ULiR (*p* value)	SCT (R/*p* value)	ULiR (*p* value)	SCT (R/*p* value)	ULiR (*p* value)
Number variation (range)						
Haemoglobin	0.000/1.000	0.7375	0.047/0.737	0.7835	0.024/0.867	0.0609
Leukocytes	0.101/0.471	0.4833	0.256/0.064	0.1695	0.162/0.247	0.1122
Neutrophils	0.125/0.372	0.4689	0.237/0.088	0.0969	0.183/0.190	0.0740
Lymphocytes	0.071/0.613	0.2391	0.118/0.401	0.9156	0.073/0.605	0.1955
Platelets	−0.170/0.223	0.3899	0.063/0.653	0.6527	−0.350/0.806	0.9802
Creatinine	−0.055/0.698	0.7700	0.105/0.459	0.5176	0.290/0.037	0.3827
Creatinine clearance	0.009/0.951	0.9380	0.096/0.496	0.7957	0.259/0.063	0.3884
	SCT (R/*p* value)	ULoR (*p* value)	SCT (R/*p* value)	ULoR (*p* value)	SCT (R/*p* value)	ULoR (*p* value)
Severity by grade						
Anaemia	0.073/0.601	0.3811	0.141/0.315	0.4889	0.025/0.858	0.3717
Leukopenia	0.041/0.771	0.8971	−0.039/0.784	0.5143	0.061/0.663	0.7397
Neutropenia	−0.007/0.960	0.9863	−0.067/0.632	0.3471	0.047/0.741	0.7066
Lymphopenia	0.071/0.613	0.4500	0.118/0.401	0.2684	0.073/0.605	0.7810
Thrombocytopenia	0.038/0.786	0.3511	−0.067/0.631	0.2847	−0.020/0.889	0.4774
Increase in serum creatinine	−0.069/0.627	0.6263	0.200/0.155	0.9527	0.325/0.019	0.1462
Reduction in creatinine clearance	−0.100/0.483	0.4715	0.249/0.078	0.9217	0.276/0.050	0.7730
Nausea	−0.065/0.642	0.6791	−0.124/0.377	0.5203	0.009/0.949	0.7742
Vomiting	−0.101/0.471	0.4140	−0.154/0.270	0.7819	−0.024/0.866	0.9087
Diarrhoea	−0.195/0.161	0.1685	−0.103/0.463	0.3765	−0.044/0.754	0.6894

*SCT* Spearman Correlation test, *R* correlation coefficient, *ULiR* Univariate Linear Regression, *ULoR* Univariate Logistic Regression. For ULoR, *p* values were only showed, because all values were > 0.05, odds ratio and 95% confidence intervals were not showed; anaemia, grade 0 *versus* grade 1 *versus* grades 2 + 3; leukopenia, neutropenia and thrombocytopenia, grade 0 *versus* grades 1 + 2 + 3 + 4; lymphopenia, grades 0 + 1 *versus* grade 2 *versus* grades 3 + 4; increase in serum creatinine, grade 0 *versus* grades 1 + 2; reduction in creatinine clearance, grade 0 *versus* grade 1 *versus* grades 2 + 3; nausea and vomiting, grade 0 *versus* grade 1 *versus* grades 2 + 3; diarrhoea, grade 0 *versus* grades 1 + 2 + 3

## Discussion

In the present study, we attempted to elucidate the relationship between ADRs and cisplatin excretion in the urine of patients undergoing a high-dose of cisplatin chemotherapy and radiotherapy for head and neck cancer, an issue that has not been demonstrated in literature yet. The pattern of ADRs and excretion of urinary cisplatin was studied along with the days of occurrence of gastrointestinal ADRs.

The mean concentration of haemoglobin in the patients’ blood before treatment was below the reference value for men (14 g/dL). There is a relation between haematological abnormalities and malignancies, including anaemia, which may be present in 3.3–29.2% of patients with solid tumours before treatment [25]. After the first chemotherapy cycle, there was a significant reduction in the mean value of haemoglobin and 81.4% of patients had anaemia. This is in agreement with the results of a study by de Castro et al. [26], in which 73% of patients treated with three cisplatin cycles and radiotherapy had anaemia (50% grades 1 and 2; 23% grades 3 and 4); 47% of the patients required red blood cell transfusion to maintain haemoglobin levels above 10 g/dL during treatment.

Some patients had leukocyte, neutrophil and platelet counts above the reference values before treatment, although the average was within the normal range. In Chinese patients with solid tumours, thrombocytosis and leukocytosis before treatment were present in 4–25.6% and 2.1–9.7%, respectively; these percentages are higher in the occident [25]. As observed in oncology patients, cancer can cause chronic neutrophilia [27]. After the first cycle of chemotherapy, there was a significant reduction in these parameters as well as lymphocytes. These changes are expected due to the myelotoxic effects of cisplatin on the bone marrow [28]. Similar to our results, the study by de Castro et al. [26], reported that 63% of patients had lymphopenia, 32% neutropenia, 29% leukopenia and 13% thrombocytopenia. However, there was a higher frequency of leukopenia, lymphopenia and thrombocytopenia and lower frequency of neutropenia in our cohort.

The severity (grade) and numeric value of haemoglobin levels did not correlate with leukocytes and platelets, and the same was observed for neutrophils and lymphocytes. As previously mentioned, the main mechanism for reducing these parameters by cisplatin is bone marrow aplasia; however, these results suggest that the response to aplasia could vary with the cell line.

In the present study, we observed a significant increase in serum creatinine after treatment, which characterises the acute nephrotoxicity caused by cisplatin. Arunkumar et al. [29] also evaluated the effect of this antineoplastic drug (40–50 mg/m^2^ weekly for 5 cycles) on serum creatinine levels in patients with HNSCC. They observed an increase (46.6%) in serum creatinine levels after treatment (pretreatment serum creatinine: 0.73 ± 0.08 mg/dL, serum creatinine after treatment: 1.07 ± 0.19 mg/dL; *p* < 0.05). In the current study, an increase of 25% in the mean serum creatinine was observed.

The increase in creatinine was only classified as grade 1 and 2; a more severe increase in creatinine (grade 3 and 4) was not observed in our study, probably due to the vigorous hydration and administration of mannitol during the course of chemotherapy. The study by de Castro et al. [26] also reported increased creatinine in patients with grade 1 and 2 (27%), similar to our study (27.6%). The incidence is consistent with the literature; 20–41% [30] exposed to cisplatin may develop kidney dysfunction.

In comparison with other chemotherapy drugs as well as among platinum compounds, cisplatin is the strongest emesis-inducing antineoplastic drug [31]. In this study, a high prevalence of nausea and vomiting was observed; nausea was the third major ADR, and vomiting was the sixth most frequent ADR. Other studies also found nausea and vomiting among the main ADRs in patients treated with cisplatin: 60% and 67% [26], 54.9% and 41.2% [32] and 43% (nausea/vomiting) [33]. Moreover, as observed by de Castro et al., most subjects had these ADRs in grade 1 and 2 [26].

Nausea and vomiting were more frequent in the delayed phase, particularly on day 3 in our population. Kris et al. [34] analysed subjects treated with cisplatin and reported delayed emesis (mostly days 2 and 3) in most of them compared with the acute phase. Emesis is less frequent in the first 24 h, which also confirms the findings of Cohen et al. [35] who evaluated 151 cancer patients.

The urinary excretion profile of cisplatin is consistent with the findings in literature. Litterest et al. [36, 37] showed that cisplatin essentially undergoes renal excretion and approximately 50% of the drug appears in the urine within 24 h. Later, Gullo et al. [38] showed that 26.6–50% of cisplatin administered is excreted in the urine in 48 h. Siddik et al. [39] found that 52% of cisplatin is excreted in the urine within 3 days following its administration, although most of it is excreted on day 1. Only Gullo et al. conducted a pharmacokinetics study on cisplatin in humans [38]. Pharmacokinetic studies determined the plasma concentration of cisplatin per mL of blood at various times during and after infusion. It was observed that the dose administered is practically related in time 0 after infusion with the total dose administered [40–42]. Our study was not aimed to determine the kinetics of cisplatin in the blood but to compare the results with those of the studies cited, considering that 100% of the dose in the blood is the total administered; the average cisplatin administered was 153.6 ± 29.2 mg, the average blood volume in adults is 4.4 L [43] (therefore, cisplatin blood concentration was 0.035 mg/mL), and the average values of cisplatin excreted in urine in the first 12 h, 12–24 h and 24–48 h were 42%, 9.4, and 9% of dose administered, which is similar to previous studies.

There was no relationship between urinary excretion of cisplatin and its ADRs. It appears that the severity of ADRs is not linked to cisplatin clearance. The most obvious result to be expected is that less drug elimination predicts greater toxicity; however, this was not observed in the study. It would be ideal to monitor serum cisplatin; however, this is hampered by the need for blood collection several times from patients who are physically weak and anaemic.

Although no correlation was found between urinary excretion and ADRs, the study results suggest the need for more research on the predictors of cisplatin toxicity, mainly related to other pharmacokinetic parameters and pharmacogenetics. Regarding the pharmacokinetic parameter distribution, cisplatin ADRs may be influenced by the concentration of serum albumin and total proteins, considering 90% of the cisplatin in plasma is linked to proteins [7] and that it is common for cancer patients to present with hypoalbuminemia; it can be assumed that the amount of free cisplatin in the plasma of these patients will be dependent on the concentration of plasma proteins, and perhaps the ratio of plasma proteins and excretion of cisplatin/ADRs may be important. Holding et al. [21] showed that the quantity of cisplatin bound to albumin is important for its toxicity and excretion. This study did not measure albumin concentration and total protein in the blood. Moreover, some reactions common to cisplatin, such as neurotoxicity, hepatotoxicity, ototoxicity and electrolyte disturbances, have not been analysed. However, these parameters are already included in our further studies. Furthermore, ADRs may be related to genetic polymorphisms that do not necessarily affect the detoxification of cisplatin. One must also consider the possible errors in urine sample collection as a limitation of this study.

## Conclusion

The incidence of ADRs to cisplatin was high after the first cycle of chemotherapy, and haematologic (lymphopenia and anaemia) and gastrointestinal (nausea) reactions were the most frequent. Grade 1 and 2 toxicity were prevalent for most ADRs. The maximum excretion of cisplatin was observed 0–12 h after chemotherapy and the amount of cisplatin excreted could not be used to predict ADRs. Other pharmacokinetic and genetic parameters should be further studied to elucidate and prevent cisplatin ADRs.

## Abbreviations

ADRs: Adverse drug reactions
ATP: Adenosine Triphosphate
CTCAE: Common Terminology Criteria for Adverse Events
DDTC: Diethyldithiocarbamate
HNSCC: Head and Neck Squamous Cell Carcinomas
HPLC: High Performance Liquid Chromatography
KPS: Karnofsky Performance Status
R: Correlation coefficient
SCT: Spearman Correlation test
SI: Smoking index
ULiR: Univariate Linear Regression
ULoR: Univariate Logistic Regression
